# RETRACTION: Impact of Evidence‐Based Nursing on Surgical Site Wound Infection After Caesarean: A Meta‐Analysis

**DOI:** 10.1111/iwj.70439

**Published:** 2025-03-26

## Abstract

**RETRACTION:** ZhangL.‐H. , WuJ.‐L. , ZhangQ. , LiN.‐M. , and LiW.‐Y. , “Impact of Evidence‐Based Nursing on Surgical Site Wound Infection After Caesarean: A Meta‐Analysis,” International Wound Journal 21, no. 2 (2024): e14688, 10.1111/iwj.14688.PMC1096104738098219

The above article, published online on 01 February 2024, in Wiley Online Library (http://onlinelibrary.wiley.com/), has been retracted by agreement between the journal Editor in Chief, Professor Keith Harding; and John Wiley & Sons Ltd. Following an investigation by the publisher, all parties have concluded that this article was accepted solely on the basis of a compromised peer review process. The editors have therefore decided to retract the article. The authors did not respond to our notice regarding the retraction.

**RETRACTION:** L.‐H. Zhang , J.‐L. Wu , Q. Zhang , N.‐M. Li , and W.‐Y. Li , “Impact of Evidence‐Based Nursing on Surgical Site Wound Infection After Caesarean: A Meta‐Analysis,” International Wound Journal 21, no. 2 (2024): e14688, 10.1111/iwj.14688.PMC1096104738098219

The above article, published online on 01 February 2024, in Wiley Online Library (http://onlinelibrary.wiley.com/), has been retracted by agreement between the journal Editor in Chief, Professor Keith Harding; and John Wiley & Sons Ltd. Following an investigation by the publisher, all parties have concluded that this article was accepted solely on the basis of a compromised peer review process. The editors have therefore decided to retract the article. The authors did not respond to our notice regarding the retraction.

